# Lack of Association of Type 2 Diabetes Susceptibility Genotypes and Body Weight on the Development of Islet Autoimmunity and Type 1 Diabetes

**DOI:** 10.1371/journal.pone.0035410

**Published:** 2012-04-25

**Authors:** Christiane Winkler, Jennifer Raab, Harald Grallert, Anette-Gabriele Ziegler

**Affiliations:** 1 Institute of Diabetes Research, Helmholtz Zentrum München, Neuherberg, Germany; 2 Forschergruppe Diabetes e.V. at Helmholtz Zentrum München, Neuherberg, Germany; 3 Research Unit of Molecular Epidemiology, Helmholtz Zentrum München, Neuherberg, Germany; 4 Forschergruppe Diabetes, Klinikum Rechts der Isar, University of Technology Munich, Munich, Germany; The Children's Hospital of Philadelphia, United States of America

## Abstract

**Aim:**

To investigate whether type 2 diabetes susceptibility genes and body weight influence the development of islet autoantibodies and the rate of progression to type 1 diabetes.

**Methods:**

Genotyping for single nucleotide polymorphisms (SNP) of the type 2 diabetes susceptibility genes *CDKAL1*, *CDKN2A/2B*, *FTO*, *HHEX-IDE*, *HMGA2*, *IGF2BP2*, *KCNJ11*, *KCNQ1*, *MTNR1B*, *PPARG*, *SLC30A8* and *TCF7L2* was obtained in 1350 children from parents with type 1 diabetes participating in the BABYDIAB study. Children were prospectively followed from birth for islet autoantibodies and type 1 diabetes. Data on weight and height were obtained at 9 months, 2, 5, 8, 11, and 14 years of age.

**Results:**

None of type 2 diabetes risk alleles at the *CDKAL1*, *CDKN2A/2B*, *FTO*, *HHEX-IDE*, *HMGA2*, *IGF2BP2*, *KCNJ11*, *KCNQ1*, *MTNR1B*, *PPARG* and *SLC30A8* loci were associated with the development of islet autoantibodies or diabetes. The type 2 diabetes susceptible genotype of *TCF7L2* was associated with a lower risk of islet autoantibodies (7% vs. 12% by age of 10 years, P = 0.015, P_corrected_ = 0.18). Overweight children at seroconversion did not progress to diabetes faster than non-overweight children (HR: 1.08; 95% CI: 0.48–2.45, P>0.05).

**Conclusions:**

These findings do not support an association of type 2 diabetes risk factors with islet autoimmunity or acceleration of diabetes in children with a family history of type 1 diabetes.

## Introduction

Type 1A diabetes and type 2 diabetes are etiologically different forms of insulin deficiency [1]. Type 1 diabetes is a chronic autoimmune disease in which selective destruction of the pancreatic islet beta cells leads to a marked insulin deficit [2]. Autoimmunity is evidenced by autoantibodies against specific islet proteins and can precede disease by many years [2]. Type 2 diabetes is characterized by insulin resistance and impaired islet beta cell function which together result in a net inability to supply sufficient insulin to meet the body's demands and eventual beta cell loss [1]. Genetic susceptibility is mostly non-overlapping in the two forms of diabetes mellitus, but several studies have suggested that there could be contribution of risk factors from type 2 diabetes to the pathogenesis of type 1 diabetes and vice versa, leading to the concept of double diabetes [3], [4]. In particular, increasing population weight and body mass index (BMI) has been linked to increasing type 1 diabetes trends [3], [4], [5], [6]. Our previous studies in children who are offspring of patients with type 1 diabetes found no association of body weight or insulin resistance with islet autoimmunity [7]. Here, as an extension of these studies we examine associations of type 2 diabetes susceptibility genotypes on the development of autoimmunity against islet beta cells and examine the effect of these genotypes and body weight on progression to diabetes after islet autoantibody seroconversion. The findings suggest that type 2 diabetes risk factors are not a common feature of type 1 diabetes occurring in first degree relatives of patients with type 1 diabetes and do not support a significant role of these type 2 diabetes associated factors in the pathogenesis of childhood diabetes in families affected with type 1 diabetes.

## Materials and Methods

The study was performed in children from the BABYDIAB study, a longitudinal study examining the natural history of islet autoimmunity and type 1 diabetes in 1650 children born to a mother with type 1 diabetes or a father with type 1 diabetes [8]. Autoantibodies against insulin (IAA), glutamic acid decarboxylase (GADA), insulinoma antigen 2 (IA-2A) and zinc transporter 8 (ZnT8-A) were measured in samples taken at all scheduled visits (at 9 months, 2, 5, 8, 11, 14, 17 and 20 years of age), and every 6 months in children with islet autoantibodies. The median follow-up time from birth to last sample was 11.1 years (range 0.75–21.7 years), and the median follow-up from islet autoantibody seroconversion to last sample was 5.4 years (range 0.1–19.6 years). Families were asked to report occurrence of symptoms of diabetes. In children with islet autoantibodies, a yearly oral glucose tolerance test was performed. Diabetes onset was defined according to ADA criteria which include unequivocal hyperglycemia with acute metabolic decompensation, or the observation on at least two occasions of a 2-hour plasma glucose >200 mg/dL after an oral glucose challenge, or a random blood glucose >200 mg/dL if accompanied by unequivocal symptoms. Since 1997, fasting blood glucose >126 mg/dL on two occasions was added to the diabetes diagnosis criteria. The BABYDIAB study was approved by the ethical committee of Bavaria, Germany (No. 95357). All families gave written informed consent to participate in the study. Investigations were carried out in accordance with the principles of the Declaration of Helsinki, as revised in 2000.

IAA, GADA, IA-2A, and ZnT8A were determined centrally by the Institute of Diabetes Research Munich using radiobinding assays as previously described [8], [9]. The upper limit of normal for each assay was determined using Q–Q plots and corresponded to the 99^th^ percentile of control children. Offspring were considered islet autoantibody-positive when two consecutive samples collected after birth were positive. Islet autoantibody assays were evaluated by the Diabetes Autoantibody Standardization Program (DASP; laboratory 121) [10], [11], [12]. Sensitivity and specificity in the 2009 DASP workshop were 70% and 98% for IAA, 86% and 93% for GADA, 72% and 100% for IA-2A, 51% and 100% for the ZnT8A tryptophan variant, and 68% and 100% for the ZnT8A arginine variant, respectively.

Genotyping of the *CDKAL1 rs4712526*, *CDKN2A/2B rs10811661*, *FTO rs8050136*, *HHEX-IDE rs5015480*, *HMGA2 rs1122590*, *IGF2BP2 rs4402960*, *KCNJ11 rs5215*, *KCNQ1 rs2237892*, *MTNR1B rs1387153*, *PPARG rs1801282*, *SLC30A8 rs3802177* and *TCF7L2 rs7901695 SNPs* was performed with the MassARRAY system using the iPLEX™ chemistry (Sequenom, San Diego, CA, USA) as previously described [13]. The 12 loci included 9 originally reported by [14] plus 3 that were used for genotyping of the KORA study cohort [15]. To control for reproducibility, 16.3% of samples were genotyped in duplicate with discordance rate <0.5%. All SNPs were tested for deviation from Hardy-Weinberg equilibrium by means of chi-square or Fisher's exact test. DNA samples for genotyping were available from 1350 children. HLA-DRB1, HLA-DQA1 and HLA-DQB1 alleles were typed using PCR-amplified DNA and non-radioactive sequence-specific oligonucleotide probes as described previously [16]. Classification into high risk HLA genotypes was based on TEDDY study inclusion genotypes for first degree relatives as previously described [17].

Data on weight and height during follow-up at age 9 months, 2, 5, 8, 11, and 14 years of age were collected from paediatric records which were completed by trained staff at birth and by paediatricians at visits after birth. For analysis, data on height, weight and BMI at follow-up were adjusted for gender and exact age at examination and were expressed as percentiles using German reference data [18]. Low weight was defined as BMI percentile ≤10, normal weight was defined as BMI percentile between 10 and 90, overweight was defined as BMI percentile ≥90.

The probability of islet autoantibodies was estimated by Kaplan-Meier analysis. Hazards ratios (HRs) were determined using Cox's proportional hazards model. Within islet autoantibody positive children, Kaplan-Meier analysis was used to calculate the probability of progression to diabetes where follow-up time was calculated from the age when autoantibodies were first detected to the age of type 1 diabetes diagnosis, or last contact. Based on a corrected alpha value of 0.004 and a desired statistical power of 0.8, our data allowed us to detect HRs for the probability of islet autoantibodies of ≤0.77 or ≥1.34 for *TCF7L2* and of ≤0.38 or ≥5.25 for *PPARG*. For all other genes, the upper and lower limits of the respective detectable HRs were between these ranges. Differences of weight, height and BMI between children who developed islet autoantibodies and children who were islet autoantibody negative during follow-up at age 9 months, 2, 5, 8, 11, and 14 years were compared using Mann-Whitney-U Test. For comparison of BMI, weight, and hight at the age of seroconversion, a nested case–control population was selected from the 1650 offspring participating in BABYDIAB. These included 141 islet autoantibody positive children (cases) with weight, and height data at the age of islet autoantibody seroconversion. For each case, two controls were selected from the islet autoantibody negative children matching for gender and data of birth. In the control children, weight and height was obtained at the age corresponding to the age of seroconversion of the respective case. Seven of these control children did not have complete weight and height data resulting in a total of 275 controls. The distribution of BMI, weight, and height were compared using Chi-Square Test.

The statistical analysis was performed using the Statistical Package for the Social Sciences (SPSS 19.0; SPSS Inc., Chicago, IL, USA).

## Results

### Influence of the type 2 diabetes susceptibility genes on islet autoimmunity and diabetes

**Figure 1:**
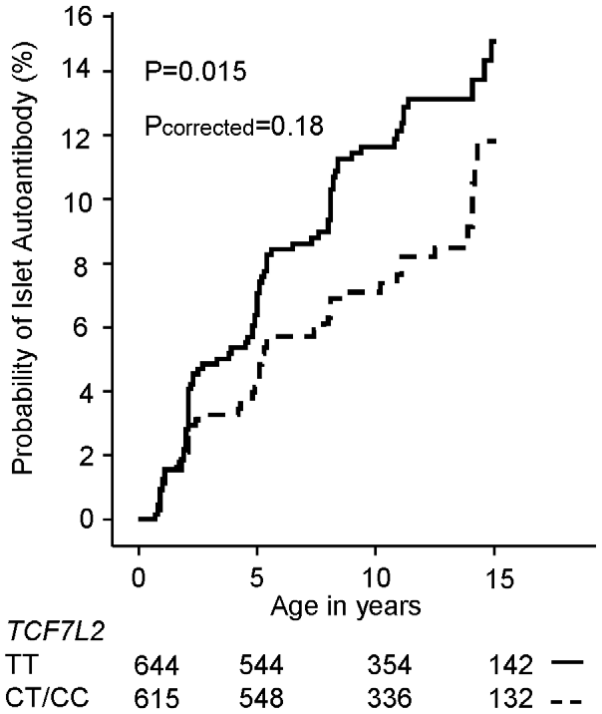
Cumulative risk for the development of autoantibodies by the *TCF7L2* genotypes. Children are grouped with respect to *TCF7L2* SNP *rs*7901695 genotype into those carrying TT genotype (solid line) and the CT or CC genotype (dashed line). Follow-up (x-axis) is from birth. Numbers below the x-axis indicate the number of autoantibody negative children remaining on follow-up with respect to age.

At the time of the analysis 152 BABYDIAB children developed islet autoantibodies. None of type 2 diabetes risk alleles at the *CDKAL1*, *CDKN2A/2B*, *FTO*, *HHEX-IDE*, *HMGA2*, *IGF2BP2*, *KCNJ11*, *KCNQ1*, *MTNR1B*, *PPARG* and *SLC30A8* loci were associated with the development of islet autoantibodies in our cohort (Table S1). The type 2 diabetes C allele of *TCF7L2* (rs7901695) was associated with a lower risk of islet autoimmunity compared to the TT genotype (HR: 0.66; 95% CI: 0.47–0.94; P = 0.022), but significance was lost after correcting for multiple comparisons (P_corrected_ = 0.264). The cumulative risk of developing islet autoantibodies by age 10 years was 7% (95% CI: 5–9%) in children carrying the *TCF7L2* CT or *TCF7L2* CC genotype compared to 12% (95% CI: 10–14%) in children carrying the type 2 diabetes protective *TCF7L2* TT genotype (P = 0.015; P_corrected_ = 0.18) (Figure 1). The observed lower risk was unaffected by adjustment for *HLA* risk genotype (HR: 0.63; 95% CI: 0.44–0.89; P = 0.009; P_corrected_ = 0.108). 21% of the cohort had high risk HLA genotypes. When selecting for this high risk population, the cumulative risk of developing islet autoantibodies by age 10 years was not different between children carrying the *TCF7L2* CT or *TCF7L2* CC genotype (17%; 95% CI: 11–23%) and children carrying the type 2 diabetes protective *TCF7L2* TT genotype (22%, 95% CI: 14–30%); (P = 0.321); HR 1.23, 95%CI: 0.75–2.2 (P = 0.360).

Among 152 islet autoantibody-positive children, 56 developed diabetes (median, 4.08 years after their first islet-positive sample). None of the type 2 diabetes susceptibility genotypes at the *TCF7L2*, *CDKAL1*, *CDKN2A/2B*, *FTO*, *HHEX-IDE*, *HMGA2*, *IGF2BP2*, *KCNJ11*, *KCNQ1*, *MTNR1B*, *PPARG* and *SLC30A8* loci were associated with progression to type 1 diabetes (data not shown).

### Weight, Height and BMI at islet autoantibody seroconverion and in relation to diabetes progression

**Figure 2:**
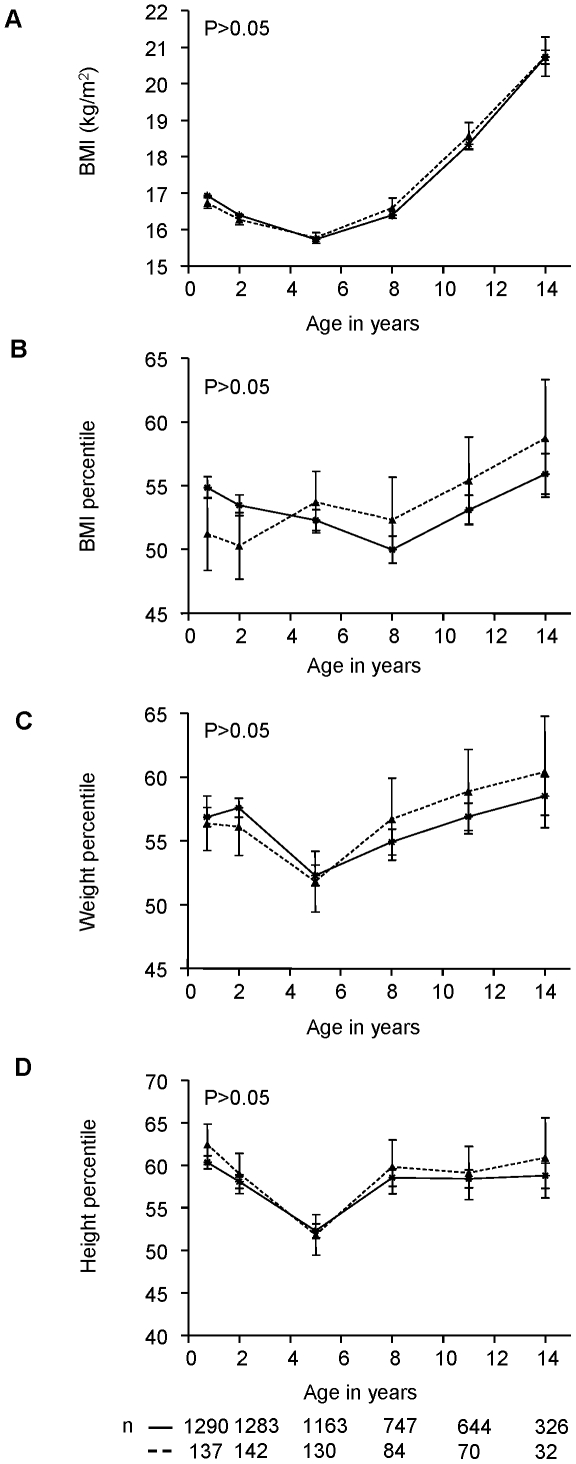
BMI, weight and height between islet autoantibody positive and negative children. Comparison of BMI (A), BMI percentile (B), weight percentile (C) and height percentile (D) between islet autoantibody positive children (dashed line) and islet autoantibody negative children (solid line) during follow-up at age 9 months, 2, 5, 8, 11, and 14 years. Values are expressed as mean ± SEM. Numbers below the x-axis indicate the number of children with available data at the respective age.

At the time of islet autoantibody seroconversion 16 antibody positive children (11.3%) were overweight (≥90 BMI percentile), and 13 (9.2%) were underweight (≤10 BMI percentile). The distribution was similar to the 275 matched islet autoantibody negative children (12.9% overweight, 9.5% underweight). No difference between weight, height and BMI was found during follow-up between children who developed islet autoantibodies and children who were islet autoantibody negative (Figure 2).

**Figure 3:**
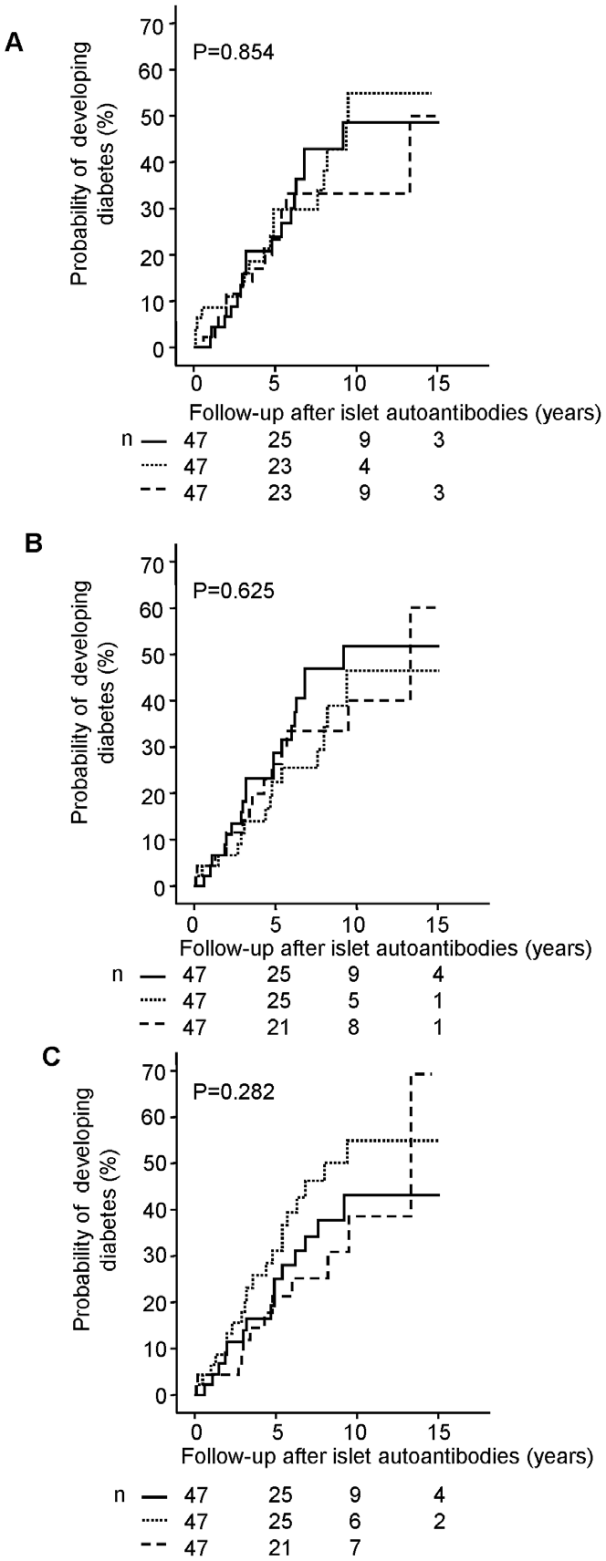
Cumulative risk for the progression from islet autoantibody seroconversion to type 1 diabetes. Cumulative risk of type 1 diabetes is shown in 141 children with islet autoantibodies in relation to BMI percentile (A), weight percentile (B) and height percentile (C) at the time of islet autoantibody seroconversion. Children are divided into 3 groups: lowest tertile (dashed line), second tertile (dotted line) and highest tertile (solid line). Follow-up (x-axis) is from the age of islet autoantibody seroconverison. Numbers below the x-axis indicate the number of diabetes-free children remaining on follow-up.

The islet autoantibody children who were overweight did not progress to diabetes faster than the remaining children (HR: 1.08; 95% CI: 0.48–2.45, P>0.05; Figure S1). Likewise, no difference in the rate of progression was observed when children were categorized into tertiles of BMI, weight, or height at islet autoantibody seroconversion (Figure 3).

## Discussion

This study examined the contribution of type 2 diabetes associated risk factors to the development of islet autoimmunity and type 1 diabetes in first degree relatives of patients with type 1 diabetes. Consistent with the lack of association of type 2 diabetes susceptibility genes with type 1 diabetes [19], [20], children with type 2 diabetes susceptible genotypes did not have an increased risk of developing islet autoantibodies and these genotypes did not increase the rate of progression to type 1 diabetes in islet autoantibody positive children. Indeed, the type 2 diabetes risk genotypes of *TCF7L2* rather decreased the likelihood of developing islet autoantibodies, but not in children with a high risk HLA genotype.

A number of the type 2 diabetes susceptibility genes are associated with increased growth rate and weight [21], [22], [23], [24]. Thus, despite substantial discussion on a link between increasing weight trends and increased type 1 diabetes development [5], [6], the genetic data which is based on a limited set of known susceptibility gene regions and a cohort of restricted sample size, neither support the concept of common pathogenetic mechanisms of type 1 diabetes and type 2 diabetes, nor an increase in type 1 diabetes risk in the presence of type 2 diabetes risk factors. Consistent with this, our study found that obesity was infrequent in German first degree relatives who developed islet autoantibodies. Moreover, although our study had a limited opportunity to determine whether obesity increased the rate of progression to diabetes in these children, weight, height and BMI had no influence on the development of type 1 diabetes in islet autoantibody positive children.

Overall, the findings indicate that mechanisms involved in type 2 diabetes pathogenesis are unlikely to have important contributions to the development of autoimmune diabetes in children from affected families. These findings may not pertain to type 1 diabetes in older subjects, as well as diabetes in people from unaffected families.

## Supporting Information

Figure S1 **Cumulative risk for the progression from islet autoantibody seroconversion to type 1 diabetes.** Cumulative risk is shown for BMI percentile in children at the time of islet autoantibody seroconversion. Children are divided into 3 groups: ≥90^th^ percentile (solid line), between 10^th^ and 90^th^ percentile (dotted line) and ≤10^th^ percentile (dashed line). Follow-up (x-axis) is from the age of islet autoantibody seroconverison. Numbers below the x-axis indicate the number of diabetes-free children remaining on follow-up.
(TIFF)

Table S1 **Gene associations with development of islet autoimmunity in the BABYDIAB cohort.**
(DOC)
