# Supplementary figures and images for: Lack of Association of Type 2 Diabetes Susceptibility Genotypes and Body Weight on the Development of Islet Autoimmunity and Type 1 Diabetes
Source: PLoS One. 2012 Apr 25;7(4):e35410. doi: 10.1371/journal.pone.0035410 (PMC3338842; doi:10.1371/journal.pone.0035410)

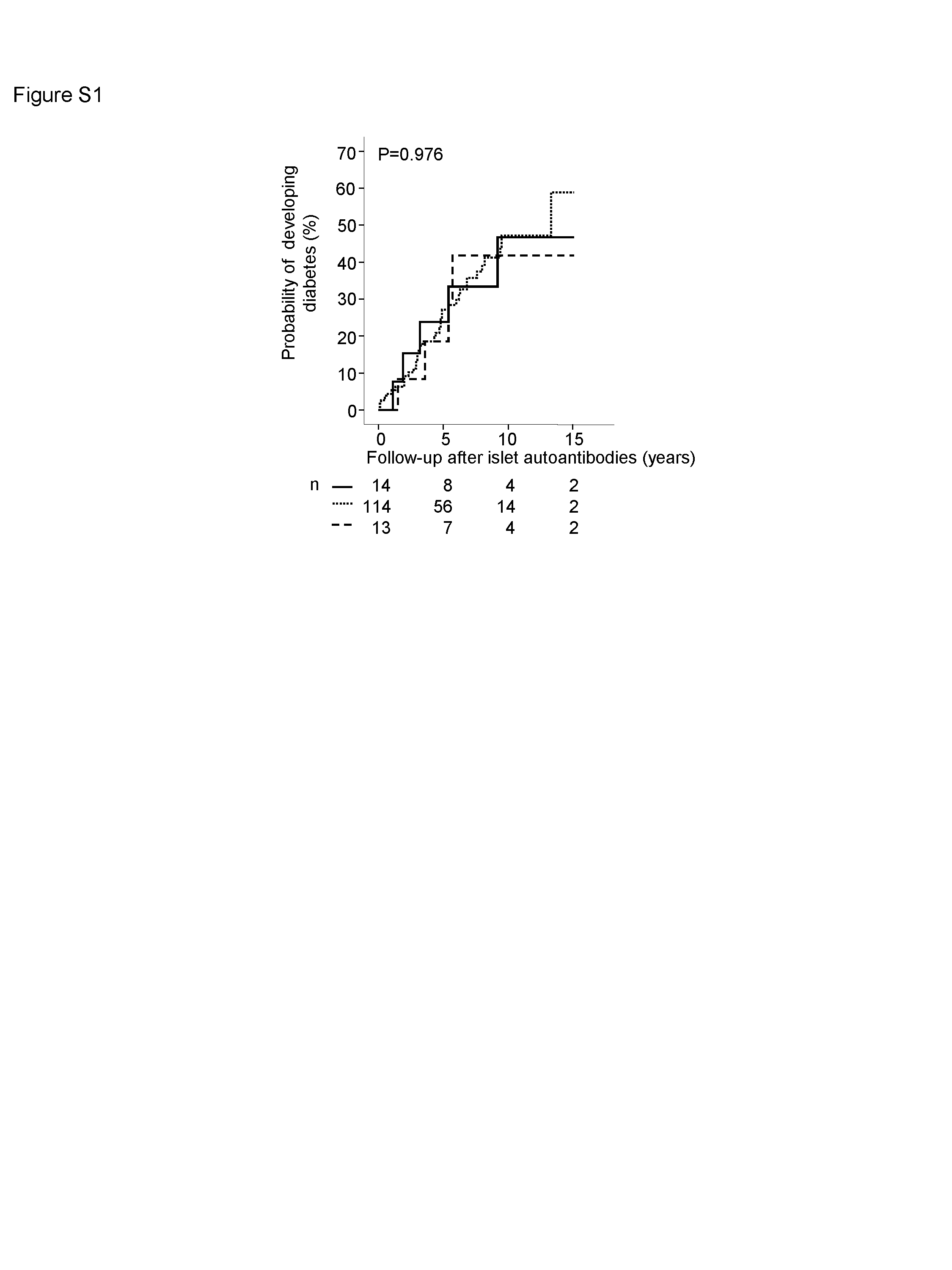

Supplement: Figure S1 — Cumulative risk for the progression from islet autoantibody seroconversion to type 1 diabetes. Cumulative risk is shown for BMI percentile in children at the time of islet autoantibody seroconversion. Children are divided into 3 groups: ≥90th percentile (solid line), between 10th and 90th percentile (dotted line) and ≤10th percentile (dashed line). Follow-up (x-axis) is from the age of islet autoantibody seroconverison. Numbers below the x-axis indicate the number of diabetes-free children remaining on follow-up. (TIFF) [file pone.0035410.s001.tif]
