# Supplementary material for: Lack of Association of Type 2 Diabetes Susceptibility Genotypes and Body Weight on the Development of Islet Autoimmunity and Type 1 Diabetes
Source: PLoS One. 2012 Apr 25;7(4):e35410. doi: 10.1371/journal.pone.0035410 (PMC3338842; doi:10.1371/journal.pone.0035410)
Supplement: Table S1 — Gene associations with development of islet autoimmunity in the BABYDIAB cohort. (DOC) [file pone.0035410.s002.doc]

| **Gene, SNP** | **Genotype** | **Frequency (%)** | | | **Hazard Ratio*** | **P value** |
| --- | --- | --- | --- | --- | --- | --- |
|  |  | **Total cohort** | **AB+** | **AB-** |  |  |
| *TCF7L2 rs7901695* | CC/CT | 48.8 | 39.2 | 49.8 | 0.66 (0.47-0.94) | 0.022^§^ |
| n = 1259^†^ | TT | 51.2 | 60.8 | 50.2 | Reference |  |
| *CDKAL1 rs4712526* | AA/AT | 55.5 | 54.5 | 55.6 | 0.96 (0.68-1.35) | 0.80 |
| n = 1329 | TT | 44.5 | 45.5 | 44.4 | Reference |  |
| *CDKN2A/B rs10811661* | TT/CT | 97.1 | 94.9 | 97.4 | 0.59 (0.28-1.26) | 0.17 |
| n = 1346 | CC | 2.9 | 5.1 | 2.6 | Reference |  |
| *FTO rs8050136* | AA/CA | 67.3 | 70.8 | 66.9 | 1.19 (0.82-1.71) | 0.37 |
| n = 1335 | CC | 32.7 | 29.2 | 33.1 | Reference |  |
| *HHEX-IDE rs5015480* | CC/CT | 84.2 | 84.3 | 84.2 | 1.01 (0.63-1.60) | 0.98 |
| n = 1321 | TT | 15.8 | 15.7 | 15.8 | Reference |  |
| *HMGA2* *rs1122590* | GG/AG | 20.1 | 20.3 | 20.1 | 1.01 (0.67-1.52) | 0.97 |
| n = 1349 | AA | 79.9 | 79.7 | 79.9 | Reference |  |
| *IGF2BP2* *rs4402960* | TT/GT | 51.3 | 51.4 | 51.3 | 1.03 (0.74-1.44) | 0.85 |
| n = 1334 | GG | 48.7 | 48.6 | 48.7 | Reference |  |
| *KCNJ11 rs5215* | CC/TC | 61.3 | 59.4 | 61.5 | 0.93 (0.66-1.30) | 0.66 |
| n = 1342 | TT | 38.7 | 40.6 | 38.5 | Reference |  |
| *KCNQ1 rs2237892* | CC/CT | 72.7 | 72.3 | 72.8 | 0.95 (0.66-1.39) | 0.81 |
| n = 1332 | TT | 27.3 | 27.7 | 27.2 | Reference |  |
| *MTNR1B rs1387153* | TT/TC | 48.8 | 48.0 | 50.0 | 0.85 (0.43-1.68) | 0.64 |
| n = 1350 | CC | 51.2 | 52.0 | 50.0 | Reference |  |
| *PPARG rs1801282* | CC/CG | 98.3 | 97.8 | 98.4 | 0.77 (0.25-2.43) | 0.66 |
| n = 1350 | GG | 1.7 | 2.2 | 1.6 | Reference |  |
| *SLC30A8 rs3802177* | CC/CT | 90.8 | 88.8 | 91.0 | 0.85 (0.60-1.46) | 0.56 |
| n = 1312 | TT | 9.2 | 11.2 | 9.0 | Reference |  |

**Table S1:** Gene associations with development of islet autoimmunity in the BABYDIAB cohort

*Hazards ratios (95% confidence interval) are shown for the homozygous and heterozygous expected susceptible genotypes versus protective genotypes;

^†^Number with successful genotype.

^§^P =0.264 (adjusted for multiple comparisions)

AB+: islet autoantibody positive, AB-: islet autoantibody negative
